# The Effect of Diet Acid Detergent Fiber Content on Finishing Lamb Meat Fatty Acid Composition, Tenderness and Stability

**DOI:** 10.1002/fsn3.71542

**Published:** 2026-02-15

**Authors:** Ockert Bernard Einkamerer, Abraham Vlok Ferreira, Michael Denis Fair, Arnold Hugo

**Affiliations:** ^1^ Department of Animal Science University of the Free State Bloemfontein Republic of South Africa; ^2^ Technical Executive Molatek Malelane Republic of South Africa

**Keywords:** fatty acid, meat color, soybean hulls, wether, wheat bran

## Abstract

The effect of incrementally increasing the acid detergent fiber (ADF) content in low fiber finishing diets on meat fatty acid (FA) composition, tenderness and stability of finishing South African Mutton Merino (SAMM) wether lambs was investigated. Four dietary treatments were formulated, only differing in ADF content, namely the control (CON), ADF1, ADF2 and ADF3 at 46.8, 59.3, 63.8, and 79.9 g ADF/kg dry matter (DM), respectively. All lambs were slaughtered at a commercial abattoir at the end of a production study of 67 days. Loin chops were evaluated for FA composition, stability and tenderness. Only the α‐linolenic acid (CON vs. ADF1 and ADF3) and conjugated linoleic acid (CLA c9,t11: CON vs. ADF1 and ADF2) composition of lamb muscle tissue was affected by ADF content, with both the lowest in the CON treatment. The oleic acid, total monounsaturated fatty acid (MUFA) and lipid omega‐6 (n‐6): omega‐3 (n‐3) ratio decreased, whereas linoleic acid, α‐linolenic acid, CLAc9,t11, total polyunsaturated fatty acid (PUFA), n‐6 and n‐3 content, as well as PUFA:SFA ratio were increased following an increased ADF content. *Longissimus* color stability (apart from day 2 L* and day 8 b* values) and shear force were left unaffected. It was concluded that the ADF content of a low fiber finishing diet fed to SAMM lambs did affect meat FA composition. The n‐6:n‐3 ratio was favorably affected by a high ADF content and more research is needed in this regard.

## Graphical abstract

Increasing the acid detergent fiber (ADF) content in low‐fiber finishing diets altered the fatty acid composition of South African Mutton Merino lamb meat, mainly by increasing α‐linolenic acid, CLA (c9,t11), total n‐3, and PUFA content. Muscle fatty acid composition was less affected than subcutaneous adipose tissue, where higher ADF increased unsaturated and n‐3 fatty acids while improving the n‐6:n‐3 ratio. Meat tenderness and color stability remained unaffected, though the n‐6:n‐3 ratio exceeded the optimal 4:1 threshold.

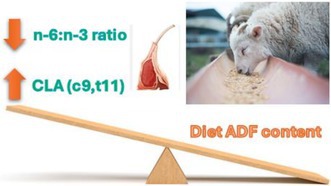

## 1 Introduction

Meat is an excellent source of protein, phosphorus, zinc, and iron (Bohrer 2017). Tissue lipids of ruminants are, however, more saturated compared to those of monogastric animals (French et al. 2000; Jenkins et al. 2008). The less favorable fatty acid (FA) composition of ruminant meat in relation to human health (Wood et al. 2003) opens the possibility of favorably improving its quality, especially polyunsaturated fatty acids (PUFA) (Wachira et al. 2002; Howe et al. 2006; Scollan et al. 2006). A high intake of saturated fatty acids (SFAs) is associated with increased risk of lifestyle‐related diseases such as higher blood cholesterol levels and atherosclerosis (Junkuszew et al. 2020). However, both SFAs and unsaturated fatty acids (UFA) are essential for human health (Hathwar et al. 2012).

Oleic acid (C18:1c9) is the major UFA in ruminant meat (Enser 1984), and lipids of ruminant origin are among the richest natural sources of conjugated linoleic acid (CLA) isomers (French et al. 2000; Hajji et al. 2016) and omega‐3 (n‐3) PUFAs, especially α‐linolenic acid (C18:3c9,12,15) (Wood et al. 2003). Conjugated linoleic acid isomers are almost exclusively derived from beef and dairy products (Mcguire and Mcguire 1999). These unique FAs have prompted the suggestion that foods derived from ruminants be considered functional foods (Mcguire and Mcguire 1999; Bauman et al. 1999) with human health benefits. Conjugated linoleic acid isomers are associated with a reduced risk of cancer (Mcguire and Mcguire 1999), cardiovascular disease and diabetes, as well as improving the immune system and bone strength (Wood et al. 2003). Biohydrogenation (BH) is the process that converts PUFAs to SFAs when fats are in free form (Toral et al. 2024) and contributes to the accumulation of *cis* and *trans* FA isomers in ruminant products (Fievez et al. 2007).

Meat quality is affected by the animal feeding system (Yagoubi et al. 2018). Any change in the rumen environment may lead to an alteration in the microbial population and activity, which in turn affects rumen lipid metabolism (Martin and Jenkins 2002). A low pH inhibits cellulolytic bacteria, the main rumen BH bacteria, thus influencing the quality of FAs reaching the small intestine (Martin and Jenkins 2002). Almost all individual and total FA concentrations of mixed rumen microbes are greatest from sheep fed a diet containing a low (184 g/kg) forage inclusion (Kucuk et al. 2008).

The roughage: concentrate ratio of a ruminant's diet is commonly used to alter FA composition. Fiber quality is rarely referred to, rather roughage source. Recently, Macdonald et al. (2023) proved that fiber source (lucerne hay, soybean hulls, maize stover and *Eragrostis tef* ) with comparable nutritional value (specifically NDF and ADF content) significantly affected finishing lamb muscle FA composition. Santos‐Silva et al. (2019) fed nutritionally similar high fiber diets differing in roughage source (dehydrated pelleted alfalfa, soybean hulls and dehydrated citrus and beet pulps), which affected meat (*Longissimus thoracis*) FA composition of Merino Branco crossbred rams.

The quality of fiber and related effects on rumen BH and meat FA composition is still largely unknown. No literature was found evaluating the effect of dietary ADF content on the FA composition of lamb meat. A low fiber diet of finishing lambs (low NDF content) seems to be the most effective option to increase beneficial FA content of lamb meat. Therefore, this study determined the effect of increasing dietary ADF content within a standard low fiber finishing diet (similar NDF content) on South African Mutton Merino (SAMM) meat FA composition, tenderness, and stability.

## 2 Materials and Methods

All procedures conducted during this study were approved by the Interfaculty Animal Ethics Committee for Animal Experimentation at the University of the Free State (Animal Experiment No. UFS‐AED2016/0038).

### 2.1 Experimental Animals, Trial Design and Housing

The production study consisted of 60 SAMM wether lambs and was conducted over 67 days. The SAMM is a dual‐purpose (mutton and wool) sheep breed (Neser et al. 2000) developed in South Africa from the German Merino with a high growth rate (Skele et al. 2024). All lambs with an initial live weight of 27.4 ± 3.2 kg (mean ± SD) were stratified and blocked according to descending weight and randomly allocated to four dietary treatments culminating in a randomized trial design with *n* = 15 lambs per treatment (*n* = 1 lamb per replicate). All lambs underwent a standard health and vaccination program prior to the study, as commonly practiced in the commercial feedlot sector of South Africa.

All animals were housed in pens (*n* = 1 lamb/pen; 1.404 m^2^) on elevated wooden slatted floors in a naturally ventilated building. Fresh, clean water was freely available to all the animals.

### 2.2 Experimental Diets and Trial Procedures

Four experimental diets were formulated to contain a similar nutrient composition, but with incremental increases in the ADF content. The physical and analyzed chemical composition of the experimental diets are presented in Table 1. Two by‐product feed sources, soybean hulls (high ADF) and wheat bran (low ADF), were used to formulate the ADF content of diets. Treatments were described according to ADF content in ascending order as CON (control: 46.8 g/kg ADF), ADF1 (59.3 g/kg ADF), ADF2 (63.8 g/kg ADF) and ADF3 (79.9 g/kg ADF). Some variation in analyzed nutrient chemical composition did occur. Weiss and St‐Pierre (2024) mentioned that variation in nutrient composition of ruminant feeds can be reduced but not eliminated. The authors presented data where the coefficient of variation (CV) for crude protein (CP) was 3.2%, 5.6%, and 20.6% within soybean meal, distillers, and poultry meal, respectively. The higher the inclusion of an ingredient, the greater its impact on nutrient variation (Weiss and St‐Pierre 2024). The latter authors explained that the standard deviation (SD) for the CP content of three diets containing different ratios of either soybean meal, distillers and poultry meal providing between 11% and 18% of total CP ranged from 0.41%, 0.42%, to 0.53%. The slight variation in chemical composition (CP, ether extract, Ca and P: SD of 0.66%, 0.37%, 0.11% and 0.10%, respectively) within the treatment diets in Table 1 is thus acceptable.

**TABLE 1:** Physical and chemical composition of the four experimental diets containing incremental increases in acid detergent fiber content.

	Treatment diets			
	CON	ADF1	ADF2	ADF3
Physical composition (g/kg as is)				
Maize meal	590.5	590.5	590.5	590.5
Molasses syrup	18.9	45.7	72.6	99.4
Soybean hulls	—	58.0	116.1	174.1
Wheat bran (whole)	269.9	180.0	90.0	—
Soybean meal	60.9	60.9	60.9	60.9
Urea	0.7	3.3	6.0	8.6
Soybean oil	26.6	25.0	23.3	21.6
Wheat germ oil	—	3.5	7.1	10.6
Limestone	15.0	11.5	8.0	4.5
Monocalcium phosphate	—	4.1	8.2	12.3
Ammonium chloride	10	10	10	10
Salt	5	5	5	5
Premix	2.5	2.5	2.5	2.5
Total	1000	1000	1000	1000
Analyzed chemical composition (g/kg DM)				
Dry matter	887.4	886.9	882.6	877.5
Organic matter	948.9	949.1	939.8	942.5
Crude protein	172.5	163.5	179.6	171.7
Non‐structural carbohydrate	566.8	548.5	552.2	555.9
Neutral detergent fiber	158.3	170.2	143.5	150.0
Acid detergent fiber	46.8	59.3	63.8	79.9
Hemicellulose	111.5	110.9	79.7	70.1
Ether extract	55.9	63.6	63.4	63.0
Ash	51.1	50.9	60.2	57.5
Total calcium	8.8	6.5	8.9	8.1
Total phosphorus	4.4	5.5	5.8	6.9

*Note:* The ADF content of diet dry matter (DM) for respective treatments is CON = 46.8 g/kg, ADF1 = 59.3 g/kg, ADF2 = 63.8 g/kg, ADF3 = 79.9 g/kg.

Abbreviations: ADF, acid detergent fiber; CON, control.

Since wheat bran decreased with increasing soybean hull inclusion, wheat germ oil was added by difference and calculated according to soybean oil inclusion to formulate comparable total diet lipid content and FA composition. The lipid content and FA composition of the four experimental diets are presented in Table 2.

**TABLE 2:** The lipid content and fatty acid composition of finishing diets fed to South African Mutton Merino wether lambs.

Fatty acid (% of total fatty acids)	Treatment diets			
	CON	ADF1	ADF2	ADF3
**Proximate analysis**				
Feed lipid content (% DM)	5.59	6.36	6.34	6.30
**Saturated fatty acids**				
Myristic (C14:0)	0.05	0.04	0.04	0.05
Palmitic (C16:0)	12.15	12.19	11.93	11.59
Stearic (C18:0)	3.03	3.01	3.15	3.19
**Monounsaturated fatty acids**				
Palmitoleic (C16:1c9)	0.07	0.07	0.07	0.07
Oleic (C18:1c9; n‐9)	20.56	20.67	21.41	21.26
Vaccenic (C18:1t11)	2.35	2.34	2.43	2.45
**Polyunsaturated fatty acids**				
Linoleic (C18:2c9,12; n‐6)	55.78	55.72	55.09	55.26
α‐Linolenic (C18:3c9,12,15; n‐3)	5.32	5.27	5.15	5.38
**Total fatty acids**				
Total saturated fatty acids (SFA)	15.68	15.70	15.60	15.33
Total monounsaturated fatty acids	22.98	23.08	23.92	23.78
Total polyunsaturated fatty acids (PUFA)	61.34	61.22	60.48	60.88
Total omega‐6 fatty acids (n‐6)	56.01	55.94	55.32	55.50
Total omega‐3 fatty acids (n‐3)	5.32	5.27	5.15	5.38
**Fatty acid ratios**				
n‐6: n‐3	10.53	10.62	10.73	10.32
PUFA: SFA	3.91	3.90	3.88	3.97

*Note:* The ADF content of diet dry matter (DM) for respective treatments is CON = 46.8 g/kg, ADF1 = 59.3 g/kg, ADF2 = 63.8 g/kg, ADF3 = 79.9 g/kg.

Abbreviations: ADF, acid detergent fiber; CON, control.

Treatment diets (mash) were fed to the animals as processed. No feed additives or rumen modifiers that may have affected rumen pH, microbial composition, or possibly FA BH were included in the diets. A synthetic antioxidant was included to prevent the oxidation of UFAs in the different diets and comprised a combination of butylated hydroxyanisole (BHA), butylated hydroxytoluene (BHT), ethoxyquin, and trisodium citrate.

At the onset of the production study (Day 0), the lambs underwent a standard adaptation period. Lucerne hay was provided *ad libitum* where each respective treatment diet was fed to the lambs and increased incrementally by 100 g/day/animal for 10 days. The animals were fed twice daily, at 08 h00 and at 16 h00. Feed intake was recorded on a weekly basis.

### 2.3 Feed Chemical Analysis

All feed samples were analyzed for DM (method 934.01 for chemical procedures), CP (Association of Official Analytical Chemists [AOAC] method 990.03—nitrogen content × 6.25), ash (method 942.05) and organic matter (OM) according to the Association of Official Analytical Chemists (AOAC 1990). The NSC content was determined as described by Van Soest et al. (1991). The NDF (Procedure A) analytical fraction was determined according to Van Soest et al. (1991) using an ANCOM^200/220^ Fiber Analyzer (ANCOM Technology Corp., Fairport, NY, USA). Total ADF (method 973.18) and total lipid (ether extract—EE; method 920.39) (AOAC 1990) were determined according to AOAC (1990). Calcium (method 935.13) and P (method 965.17) were determined according to the AOAC (2000). Diet hemicellulose content was calculated by subtracting ADF content from NDF (NRC 2007).

### 2.4 Meat Evaluation

At the end of the production study, all lambs were slaughtered at a commercial abattoir. Three loin chops (the left 9th to the 11th rib) from each carcass were evaluated (*n* = 15 repetitions per treatment).

#### 2.4.1 Fatty Acid Profile Determination

Total lipid from feed, *longissimus thoracis* muscle and subcutaneous fat sampled were quantitatively extracted according to the method of Folch et al. (1957). Chloroform and methanol were used in a ratio of 2:1. Total extractable intramuscular fat was determined gravimetrically from the extracted fat and expressed as percentage fat (w/w) per 100 g tissue. An antioxidant (BHT) was added at a concentration of 0.001% to the chloroform: methanol mixture. A rotary evaporator was used to dry the fat extracts under vacuum. The extracts were dried overnight in a vacuum oven at 50°C, using phosphorus pentoxide as a moisture adsorbent.

The extracted fat from the feed, muscle, and subcutaneous fat was stored in a polytop (glass vial, with push‐in top) under a blanket of nitrogen and frozen at −20°C pending FA analyses. A lipid aliquot (20 mg) of feed, muscle, and subcutaneous lipid was converted to methyl esters by base‐catalyzed transesterification with sodium methoxide (0.5 M solution in anhydrous methanol) for 2 h at 30°C—in order to avoid CLA isomerization, as proposed by Kramer et al. (2002) and Alfaia et al. (2007). Fatty acid methyl esters (FAME) from the feed, muscle, and subcutaneous lipid were quantified using a Varian 430 flame ionization gas chromatograph, with a fused silica capillary column, Chrompack CPSIL 88 (100 m length, 0.25 mm ID, 0.2 μm film thickness). Analysis was performed using an initial isothermic period (40°C for 2 min). Thereafter, the temperature was increased at a rate of 4°C/min to 230°C. Finally, an isothermic period of 230°C for 10 min followed. Fatty acid methyl esters n‐hexane (1 μL) was injected into the column using a Varian CP‐8400 Autosampler. The injection port and detector were both maintained at 250°C. Hydrogen, at 45 psi, functioned as the carrier gas, while nitrogen was employed as the makeup gas. Galaxy Chromatography Data System Software recorded the chromatograms.

Fatty acid methyl esters from the feed, muscle and subcutaneous lipid were identified by comparing retention times of FAME peaks from samples with those of accepted standards obtained from the Supelco (Supelco 37 Component Fame Mix 47885‐U, Sigma‐Aldrich Aston Manor, Pretoria, South Africa). Conjugated linoleic acid standards were obtained from Matreya Inc. (Pleasant Gap, Unites States of America). These standards included: C18:2c9,t11 and C18:2 t10,c12 isomers. All other reagents and solvents were of analytical grade and obtained from Merck Chemicals (Pty Ltd., Halfway House, Johannesburg, South Africa). Fatty acids were expressed as the proportion of each individual FA to the total of all FAs present in the sample. The following were also calculated: total SFA, total monounsaturated fatty acids (MUFA), total PUFA, total n‐3 and total n‐6 FAs. Total fatty acid data were used to calculate the following ratios: PUFA/SFA, n‐6/n‐3 and ∆^9^ desaturase index (C18:1c9/C18:0). Atherogenicity index was calculated as (C12:0 + 4 × C14:0 + C16:0)/(MUFA + PUFA) (Chilliard et al. 2003).

#### 2.4.2 Stability of Fresh and Frozen Lamb Chops

According to the methods as described by De Klerk et al. (2025), one loin chop from each lamb carcass was individually placed in a polystyrene tray containing an absorbent pad, wrapped with oxygen‐permeable polyvinyl chloride (PVC) meat stretch wrap and stored for 8 days at 4°C under fluorescent light. Chop were evaluated every second day for fresh meat stability. Another loin chop from each carcass was vacuum sealed and stored for 90 days at −18°C in the dark for frozen storage stability evaluation.

#### 2.4.3 Muscle Color

The color (L*, a* and b*‐values) of muscle was determined in triplicate using a refrigerated (4°C) loin chop over eight days (fresh sample at Day‐0, Day‐4 and Day‐8) using a Minolta chromo meter. Chroma or saturation index, which is related to color intensity of meat, was calculated according to the formula (a*2 + b*2) × 0.5 for muscle (Ripoll et al. 2011). Hue angle was also calculated according to the formula: tan‐1 (b*/a*) (Ripoll et al. 2011).

#### 2.4.4 Thiobarbituric Acid Reactive Substance Determination

A 5 g sample of muscle was removed from the middle of each loin chop on day zero, Day‐8 (refrigerated at 4°C) and Day‐90 (frozen storage at −18°C) to determine the malonaldehyde content/kg meat using the aqueous acid extraction method of Raharjo et al. (1992).

#### 2.4.5 Meat Tenderness Evaluation

The *longissimus* muscle (left 11th to 13th rib sample) prepared according to an oven‐broiling method using direct heat (AMSA 1978). Four cylindrical samples (12.5 mm core diameter) of each sample were cored parallel to the grain of the meat and sheared perpendicular to the fiber direction using a Warner Bratzler shear force device mounted on a Universal Instron apparatus (cross head speed = 200 mm/min; one shear in the center of each core). The reported value in kg represented the average of the peak force measurements of each sample.

### 2.5 Statistical Analysis

The data were analyzed as a completely randomized design using the General Linear Model (GLM) procedures of the Statistical Analysis System (SAS) program (SAS 1999). Means were compared using the LSMEANS/DIFF with treatment as fixed effects. For post hoc analysis, Tukey's honest significant difference (HSD) test was used to identify significant differences between treatment means at a 5% probability level. The description of the model used for ANOVA analysis was:

Yij=μ+ti+εij,

where *Yij* is the individual observations (dependent variable) of the *i*‐th treatment (independent variable) and the *j*‐th random error, μ is the general effect, *ti* is the effect of the i‐th treatment, *εij* is the random variation or experimental error.

The *i*‐th treatment effect (dietary NDF content) during this study was defined as: *i*_1_ = CON (46.8), *i*_2_ = ADF1 (59.3), *i*_3_ = ADF2 (63.8), *i*_4_ = ADF3 (79.9 g/kg), presented on a DM basis.

## 3 Results and Discussion

### 3.1 Fatty Acid Composition of Lamb Muscle Tissue

The effect of increasing ADF content in low fiber finishing diets on the lipid content and FA composition of SAMM wether lamb muscle tissue is presented in Table 3. There was only a single positive influence of ADF on the α‐linolenic acid content. This increase was, however, not consistent and related only to ADF1 and ADF3 compared to CON. The CLAc9,t11 content was also higher, but only for ADF1 and ADF2 compared to CON. The FA composition of muscle tissue is less influenced by diet compared to adipose tissue (Scollan et al. 2006). In ruminants, glucose is the favored substrate for lipogenic adipocytes in muscle, unlike subcutaneous adipocytes where acetate is favored (Dodson et al. 2010). Manso et al. (2009) stated that a lack of significant changes in intramuscular fat FA composition compared to subcutaneous adipose tissue could also be that during the finishing period, subcutaneous adipose tissue increases at a greater rate compared to other adipose depots. These authors acknowledged that subcutaneous fat seems to be more responsive to changes in the dietary FA supply, or changes in rumen metabolism, than intramuscular fat. The fact that intramuscular fat composition is not largely influenced owns to the fact that muscle lipids are phospholipids (membrane lipids) that determine membrane fluidity and cellular function; these are compositionally regulated and change slowly (Wood et al. 2008). In other research, the quantity of NDF was proven not to affect the CLA (C18:2c9,t11) content of South African Mutton Merino lamb muscle or adipose tissue (De Klerk et al. 2025).

**TABLE 3:** The effect of increasing acid detergent fiber content in finishing diets on the lipid content and fatty acid composition of South African Merino mutton *Longissimus thoracis* muscle tissue.

Longissimus thoracis fatty acid (% of total fatty acids)		Treatment diet means (± SD)				*p*	CV (%)
		CON	ADF1	ADF2	ADF3		
	Muscle lipid content (%)	2.82 (0.83)	3.32 (0.88)	2.80 (0.05)	2.95 (0.51)	0.1867	23.98
Saturated fatty acids	Myristic (C14:0)	2.14 (0.41)	2.41 (0.47)	2.31 (0.60)	2.10 (0.31)	0.2126	20.45
	Palmitic (C16:0)	27.12 (1.727)	28.22 (1.18)	27.66 (2.00)	27.41 (1.53)	0.3114	5.90
	Stearic (C18:0)	13.48 (1.18)	13.59 (1.03)	14.02 (1.47)	13.99 (1.31)	0.5492	9.11
Monounsaturated fatty acids	Palmitoleic (C16:1c9)	1.70 (0.27)	1.59 (0.26)	1.66 (0.38)	1.44 (0.27)	0.0965	18.59
	Oleic (C18:1c9; n‐9)	35.36 (2.56)	34.42 (2.48)	34.52 (2.05)	33.92 (2.21)	0.4094	6.77
	Vaccenic (C18:1 t11)	2.98 (0.88)	3.21 (0.49)	3.14 (0.42)	3.28 (0.49)	0.5719	19.06
Polyunsaturated fatty acids	Linoleic (C18:2c9,12; n‐6)	10.78 (2.31)	10.60 (2.13)	10.90 (2.27)	11.73 (2.37)	0.5417	20.64
	α‐Linolenic (C18:3c9,12,15; n‐3)	0.56^b^ (0.12)	0.68^a^ (0.11)	0.61^ab^ (0.10)	0.70^a^ (0.07)	0.0021	16.42
	Conjugated linoleic (C18:2c9, t11; n‐6)	0.17^b^ (0.08)	0.25^a^ (0.09)	0.24^a^ (0.09)	0.20^ab^ (0.06)	0.0345	37.79
Total fatty acids	Saturated fatty acids (SFA)	44.42 (2.13)	45.91 (2.02)	45.40 (2.03)	44.97 (2.03)	0.2480	4.54
	Monounsaturated fatty acids (MUFA)	41.21 (2.51)	40.20 (2.66)	40.25 (2.10)	39.59 (2.22)	0.3295	5.92
	Polyunsaturated fatty acids (PUFA)	14.38 (3.44)	13.89 (3.05)	14.35 (3.38)	15.43 (3.37)	0.6302	22.83
	Unsaturated fatty acids (UFA)	55.58 (2.13)	54.09 (2.01)	54.60 (2.03)	55.02 (2.03)	0.2476	3.74
	Omega‐6 fatty acids (n‐6)	13.30 (3.19)	12.78 (2.84)	13.27 (3.11)	14.21 (3.18)	0.6428	23.01
	Omega‐3 fatty acids (n‐3)	1.07 (0.32)	1.11 (0.28)	1.08 (0.34)	1.22 (0.25)	0.5016	26.55
Fatty acid ratios	n‐6: n‐3	12.85 (2.32)	11.78 (2.12)	12.59 (1.95)	11.72 (1.97)	0.3585	17.14
	PUFA: SFA	0.33 (0.09)	0.30 (0.08)	0.32 (0.09)	0.35 (0.09)	0.6086	26.35
	∆^9^ desaturase index	2.64 (0.33)	2.55 (0.33)	2.49 (0.34)	2.44 (0.26)	0.3472	12.45
	Atherogenicity index	0.53 (0.06)	0.57 (0.05)	0.55 (0.06)	0.54 (0.05)	0.2058	10.04
Long‐chain fatty acids	Arachidonic (C20:4c5,8,11,14; n‐6)	2.20 (0.89)	1.80 (0.78)	1.99 (0.86)	2.15 (0.84)	0.5742	41.39
	Eicosopentaenoic (C20:5c5,8,11,14,17; n‐3)	0.20 (0.09)	0.16 (0.09)	0.18 (0.11)	0.21 (0.09)	0.5691	50.30
	Docosapentaenoic (C22:5c7,10,13,16,19; n‐3)	0.28 (0.13)	0.25 (0.14)	0.27 (0.13)	0.30 (0.12)	0.7390	46.81
	Docosahexaenoic acid (C22:6c4,7,10,13,16,19; n‐3)	0.01 (0.03)	0.01 (0.02)	0.01 (0.05)	0.00 ± 0.02	0.8336	380.06

*Note:* The ADF content of diet dry matter (DM) for respective treatments are CON = 46.8 g/kg, ADF1 = 59.3 g/kg, ADF2 = 63.8 g/kg, ADF3 = 79.9 g/kg. ^a,b^Mean values with different superscripts in the same row differ significantly (*p* < 0.05).

Abbreviations: ADF, acid detergent fiber; CON, control; CV, coefficient of variation.

Importantly, dietary treatments within the current study contained no trace of CLA isomers. Both a decreased (Qiu et al. 2004; Xu et al. 2014) and increased rumen pH (Hur et al. 2017) cause incomplete BH. Low rumen pH specifically is the main driving force determining the establishment of *t*10 fatty acids reaching the small intestine (Santos‐Silva et al. 2019). Lower CLAc9,t11 is, for instance, observed at low rumen pH conditions (Choi et al. 2005). Hence, a high proportion of forage in diets can enrich beef with BH intermediates such as CLAc9,t11 (Vahmani et al. 2017). The CLAc9,t11 isomer can also be produced in body tissues by the ∆^9^ desaturase enzyme activity (Hur et al. 2017). It is interesting to note that lamb meat lipids have a significantly higher content of CLA compared with other ruminant meat lipids, which may indicate that the process of transesterification taking place in the rumen is much more efficient in lamb than in other ruminants (Garcia et al. 2008).

Delta‐9 desaturase is responsible for the conversion of all SFAs to its respective MUFA in fat tissues and is approximately twice as active in subcutaneous adipose tissue compared to intramuscular adipose tissue (Smith et al. 2006). This complicates the search for any significant effect of dietary treatment on all *cis* and *trans* FAs influenced by dietary treatment. The significant effect on α‐linolenic acid and CLAc9,t11 muscle composition as a result of rumen pH was not evaluated in the current study. Even though no conclusive result regarding rumen pH can be drawn, the lack of treatment effect on the ∆^9^ desaturase index in muscle tissue rejects a possible metabolic effect on its CLAc9,t11 content as well.

Manipulating rumen fermentation patterns to affect lipid metabolism through the diet seems to be sufficient to alter not just the rumen FA composition (French et al. 2000; Kucuk et al. 2001), but also those absorbed and deposited in the carcass (Bauman et al. 1999; French et al. 2000; Booyens et al. 2012; Du Toit et al. 2013; Ladeira et al. 2014). The FA composition of lipids is extensively altered by rumen microbes resulting in marked decreases of mostly UFAs, even up to 70%–90% (Chilliard 1993). The two major processes involved include lipolysis and BH (Jenkins 1993; Jenkins et al. 2008). The rate thereof in turn is directly influenced by microbial composition (Gerson et al. 1983) as instigated by feed type and composition (Gerson et al. 1983; French et al. 2000; Xu et al. 2014). The FA composition of rumen microbes is of interest in terms of microbial function and contribution of FAs to the host animal in the small intestine (Kucuk et al. 2008). Up to 30% of the FAs reaching the proximal duodenum are of microbial origin (NRC 2007).

The effect of increasing ADF content in low fiber finishing diets on the lipid content and FA composition of SAMM wether lamb subcutaneous lipid tissue is presented in Table 4.

**TABLE 4:** The effect of increasing ADF content in finishing diets on the lipid content and FA composition of SAMM subcutaneous lipid tissue (mean).

Fatty acid (% of total fatty acids)		Treatment diet means (±SD)				*p*	CV (%)
		CON	ADF1	ADF2	ADF3		
	Muscle lipid content (%)	76.38 (5.23)	76.07 (7.03)	75.08 (7.90)	78.48 (5.59)	0.5513	8.49
Saturated fatty acids	Myristic (C14:0)	3.01 (0.73)	3.14 (0.68)	3.07 (0.46)	3.07 (0.51)	0.9408	19.83
	Palmitic (C16:0)	27.59 (2.01)	28.89 (1.77)	28.18 (2.05)	28.27 (1.98)	0.3513	6.92
	Stearic (C18:0)	15.67 (2.31)	14.72 (2.04)	16.81 (2.49)	16.64 (2.61)	0.0722	14.86
Monounsaturated fatty acids	Palmitoleic (C16:1c9)	1.23 (0.32)	1.31 (0.26)	1.16 (0.18)	1.15 (0.23)	0.3016	20.86
	Oleic (C18:1c9; n‐9)	34.57^a^ (2.87)	34.09^ab^ (1.79)	33.50^ab^ (2.54)	32.11^b^ (2.65)	0.0522	7.43
	Vaccenic (C18:1 t11)	3.78 (1.06)	3.68 (0.29)	3.55 (0.20)	3.56 (0.24)	0.6906	15.86
Polyunsaturated fatty acids	Linoleic (C18:2c9,12; n‐6)	6.76^b^ (1.45)	7.08^b^ (1.17)	7.46^ab^ (1.16)	8.65^a^ (1.63)	0.0024	18.33
	α‐Linolenic (C18:3c9,12,15; n‐3)	0.62^c^ (0.11)	0.75^b^ (0.14)	0.74^bc^ (0.09)	0.95^a^ (0.14)	< 0.0001	16.05
	Conjugated linoleic acid (CLA) (C18:2c9, t11; n‐6)	0.25^b^ (0.08)	0.34^ab^ (0.11)	0.37^a^ (0.11)	0.32^ab^ (0.09)	0.0179	30.39
Total fatty acids	Saturated fatty acids (SFA)	50.98 (2.79)	51.07 (1.70)	51.78 (2.51)	51.81 (3.29)	0.7376	5.13
	Monounsaturated fatty acids (MUFA)	41.34^a^ (3.08)	40.70^ab^ (2.19)	39.61^ab^ (2.99)	38.19^b^ (3.22)	0.0239	7.25
	Polyunsaturated fatty acids (PUFA)	7.68^b^ (1.57)	8.23^b^ (1.33)	8.61^ab^ (1.20)	10.00^a^ (1.78)	0.0007	17.27
	Unsaturated fatty acids (UFA)	49.02 (2.79)	48.93 (1.70)	48.22 (2.51)	48.19 (3.29)	0.7379	5.43
	Omega‐6 fatty acids (n‐6)	7.06^b^ (1.48)	7.47^b^ (1.22)	7.87^ab^ (1.13)	9.05^a^ (1.64)	0.0017	17.60
	Omega‐3 fatty acids (n‐3)						
Fatty acid ratios	n‐6: n‐3	11.32^a^ (1.17)	9.99^bc^ (0.95)	10.68^ab^ (0.76)	9.46^c^ (0.70)	< 0.0001	8.85
	PUFA: SFA	0.15^b^ (0.03)	0.16^b^ (0.03)	0.17^ab^ (0.02)	0.19^a^ (0.04)	0.0033	18.79
	∆^9^ desaturase index	2.27 (0.53)	2.37 (0.42)	2.04 (0.39)	1.99 (0.44)	0.0747	20.65
	Atherogenicity index	0.63 (0.09)	0.66 (0.07)	0.65 (0.08)	0.66 (0.08)	0.7350	12.14
Long‐chain fatty acids	Arachidonic (C20:4c5,8,11,14; n‐6)	0.04^b^ (0.03)	0.06^ab^ (0.02)	0.04^b^ (0.03)	0.08^a^ (0.02)	0.0036	48.20
	Eicosopentaenoic (C20:5c5,8,11,14,17; n‐3)	—	—	—	—	—	—
	Docosapentaenoic (C22:5c7,10,13,16,19; n‐3)	—	—	—	—	—	—
	Docosahexaenoic acid (C22:6c4,7,10,13,16,19; n‐3)	—	—	—	—	—	—

*Note:* The ADF content of diet dry matter (DM) for respective treatments are CON = 46.8, ADF1 = 59.3, ADF2 = 63.8, ADF3 = 79.9 g/kg. ^a–c^Mean values with different superscripts in the same row differ significantly (*p* < 0.05).

Abbreviations: ADF, acid detergent fiber; CON, control; CV, coefficient of variation.

The lipid content as well as the myristic (C14:0), palmitic (C16:0) and stearic acid (C18:0) content of the adipose tissue was not affected by dietary ADF content. Oleic acid was, however, lower with a high ADF content (ADF3) compared to a low ADF content (CON). Delta‐9 desaturase is involved in the desaturation of some SFAs in the animal's tissues to produce oleic acid (De Smet et al. 2004). The concentration of oleic acid in bovine adipose tissue is therefore primarily dependent on the action of Δ^9^ desaturase activity (Smith et al. 2006) and is the rate‐limiting enzyme (Smith et al. 2002). Comparable to muscle tissue, this was not the case as the ∆^9^ desaturase index of the lipid tissue in the current study was unaffected by dietary treatment. This is despite the fact that subcutaneous adipose tissue has approximately twice the Δ^9^ desaturase catalytic activity compared to intramuscular adipose tissue (Smith et al. 2006).

In contrast, an increase in ADF content increased α‐linolenic acid content of the lipid tissue. A similar effect (*p* = 0.0021) was noted regarding the α‐linolenic acid content in muscle tissue as well (Table 3). This was only one of two similarities between muscle and subcutaneous lipid tissue (Tables 3 and 4, respectively). The linoleic acid content was also higher for the ADF3 compared to CON treatment, whereas the CLAc9,t11 of ADF2 was higher compared to CON. The significant effect on both the muscle and subcutaneous CLAc9,t11 content of lamb meat occurred despite the dietary treatment containing no CLAc9,t11 (Table 2).

Rumen BH is a set of microbial biochemical processes by which dietary UFAs are isomerized, hydrogenated and ultimately converted to stearic acid (Santos‐Silva et al. 2019). A possible cause for increased lipid CLA content could be related to an incomplete BH affected by both a decreased (Qiu et al. 2004; Xu et al. 2014) or increased rumen pH (Hur et al. 2017). The resultant CLAc9,t11 isomer arises from microbial hydrogenation of dietary linoleic acid in the rumen (Hajji et al. 2016) via an isomerase enzyme produced by bacteria (Harfoot and Hazlewood 1997). This is followed by the slower conversion to vaccenic acid (C18:1 t11) as an intermediate (Harfoot and Hazlewood 1997). Lower CLAc9,t11 is, for instance, observed at low rumen pH conditions (Choi et al. 2005). Another influence on lipid CLA content is the activity of the ∆^9^ desaturase enzyme in body tissues (Xu et al. 2014) by converting vaccenic acid to CLAc9,t11 (Corl et al. 2001; Smith et al. 2006; Carvalho et al. 2014; Hur et al. 2017). The majority of CLAc9,t11 is produced within animal tissues (Ishlak et al. 2015), hence less of a diet influence. Delta‐9 desaturase in animal tissue is also the terminal step in the desaturation of stearic acid into oleic acid as well (De Smet et al. 2004).

According to Santos‐Silva et al. (2019) extensive alteration in rumen BH pathways must be the result of extensive changes in rumen microbiota (due to diet and pH alterations), but the exact nature of such changes remains to be established. Therefore, the rate of fiber degradation in the rumen, effectively referring to fiber quality, should affect the rumen BH pathways. Replacing soybean hulls NDF (highly digestible) by alfalfa NDF (less digestible and slower degradation rate) in isoNDF diets supplemented with 6% soybean oil fed to crossbred male lambs born from Merino Branco ewes, for example, led to a linear decrease of t10–18:1 and t10,c12–18:2 and to a linear increase in t11–18:1 and c9,t11–18:2 within lipid tissue (Santos‐Silva et al. 2019).

High dietary hemicellulose and pectin content may induce shifts in the bacterial caecotroph populations of rabbits (Papadomichelakis, Karagiannidou, et al. 2010). It is therefore plausible that as the hemicellulose content decreased from CON to ADF3 in the current study (Table 1), the microbial composition favoring this carbohydrate as an energy source probably decreased in the rumen of sheep. The specific organism is *Prevotella ruminicola* (Matsui et al. 1998), which plays a significant role in the metabolism of proteins and peptides in the rumen (Wallace et al. 1997). No reference to its role as a rumen BH organism was, however, established in literature. Hemicellulolytic species are also not necessarily absolute hemicellulose degraders (Matsui et al. 1998). Amylolytic bacteria can ferment hemicellulose to propionate (Cheeke 2005), and the high‐concentrate diet of the current study further complicates this assumption. More research to determine the same dietary treatments' effect on rumen microorganism composition and FA BH in vitro and in vivo is required in this regard.

There was also a lack of treatment effect on the total SFA content of lamb adipose tissue (Table 4). In contrast, the total PUFA adipose tissue content significantly increased following a high dietary ADF content in ADF3, compared to CON and ADF1. This result followed a similar significant tendency regarding the linoleic and α‐linolenic acid adipose tissue content. Increased total PUFA can be attributed to the higher incorporation of linoleic acid in the same tissue (Papadomichelakis, Anastasopoulos, et al. 2010). In contrast, a lack of significant effect on the total UFA content of lamb lipid tissue was unexpected due to the significant effect of diet ADF content on the oleic acid, linoleic acid, α‐linolenic, CLAc9,t11, as well as total MUFA and PUFA content. A possible explanation could be the irregular effect on individual PUFAs following dietary treatment, as well as a decreased oleic acid and total MUFA content following a high ADF inclusion (ADF3 compared to CON). A high diet ADF content (ADF3) increased the total PUFA content compared to CON and ADF1 of lamb lipid tissue. Polyunsaturated fatty acids are, however, not commonly synthesized by rumen bacteria and are likely the result of exogenous uptake of preformed FAs, or a possible decrease in the BH (Jenkins 1993). A low fiber diet high in ADF PUFA lipid tissue content.

Lipolytic and hydrogenation rates vary with forage quality (Gerson et al. 1986; Jenkins 1993) where diminished rates of lipolysis and BH are caused by the increasing forage maturity (Jenkins 1993). In other words, fiber quality as affected by the rate of fiber degradation in the rumen can affect rumen BH pathways (Santos‐Silva et al. 2019). Hence, the rate of in vitro lipolysis and BH rates decreases with an increase in pasture age (ryegrass regrowth stage) (Gerson et al. 1986). Not all forages, however, behave in the same way when exposed to hydrogenating bacteria. More extensive rumen BH of α‐linolenic acid in grass silage compared to white or red clover silage was, for example, noted in literature (Dewhurst et al. 2006). The reason for the reduced BH rate of white clover silage may be due to a much higher rumen passage rate, which reduces exposure of forage lipids to lipases and BH in the rumen (Dewhurst et al. 2006). Ponnampalam et al. (2001) assessed the influence of low‐quality hay (oats hay) versus medium‐quality hay (lucerne hay) on the FA composition of lamb meat. In addition, molar proportions of FAs in the subcutaneous adipose tissue of wethers shifted the proportions of SFAs towards UFAs with increasing dietary energy levels (Casey and Webb 1995). The highly digestible fiber content of rabbit diets, specifically the hemicellulose and pectin content, significantly decreased MUFA but increased PUFA content of muscle tissue (*Longissimus lumborum* and *Biceps femoris*). The response reported by Papadomichelakis et al. (Papadomichelakis, Karagiannidou, et al. 2010; Papadomichelakis, Anastasopoulos, et al. 2010) on rabbit muscle tissue was similar to the effect of increasing dietary ADF content (decreasing hemicellulose content) on lipid MUFA and PUFA content of lamb adipose tissue in the current study (Table 4).

Macdonald et al. (2023) found that the n‐3 fatty acids α‐linolenic and eicosapentaenoic (C20:5c5,8,11,14,17; n‐3) acid, as well as total n‐3, increased, whereas the n‐6:n‐3 ratio favorably decreased after lucerne hay inclusion in finishing diets of lambs, compared to soybean hulls, maize stover, and *Eragrostis tef*. Macdonald et al. (2023) included balanced diets for total NDF and ADF content; hence, only the type of roughage source differed. Santos‐Silva et al. (2019) also fed nutritionally similar diets differing in roughage source only (dehydrated pelleted alfalfa, soybean hulls, and dehydrated citrus and beet pulps) to Merino Branco crossbred rams, which, in contrast, had no effect on the sum of n‐3 *Longissimus thoracis* FA composition. The diets in the Santos‐Silva et al. (2019) trial were, however, high‐fiber diets and not comparable to the study by Macdonald et al. (2023), or the current study. Limited research exists in this regard.

The total n‐6 (ADF3 compared CON and ADF1) and n‐3 (ADF1, ADF2 and ADF3 compared CON) content of lamb lipid tissue increased following increased dietary ADF content. The significant decrease (*p* < 0.0001) in the n‐6:n‐3 ratio of subcutaneous lipid tissue was accounted for by an increase in the total n‐3 content as ADF increased. This ratio is primarily the balance between n‐3 PUFA formed from α‐linolenic acid and n‐6 PUFA formed from linoleic acid (Wood et al. 2003). In addition, the higher ADF content effecting a subcutaneous tissue increase in PUFA as well as no effect on SFA content resulted in a significant increase in PUFA:SFA (*p* = 0.0033).

Not only individual FAs, but the ratio is important for human health. The PUFA:SFA ratio of 0.45 (Cooke et al. 2004; Demirel et al. 2006), or 0.40 and higher (Wood et al. 2003; Webb and O'Neill 2008) is nutritionally important. A lesser PUFA:SFA value of 0.50 to 0.80 is also proposed (McDonald et al. 2011). Lamb and beef PUFA:SFA ratio is very low at 0.10 (Scollan et al. 2006). The average PUFA:SFA ratio of lamb subcutaneous lipid varied between 0.15 (CON) to 0.19 (ADF3) (Table 4), which was still not ideal, but higher than 0.10. An n‐6:n‐3 ratio of less than 4:1 (Cooke et al. 2004; McDonald et al. 2011; Lestingi et al. 2015) is also important for human health. According to Wijendran and Hayes (2004), an n‐6:n‐3 ratio of approximately 6:1 is adequate. The primary concern is the effect on α‐linolenic and linoleic acid content of ruminant products (Wood et al. 2003). The n‐6:n‐3 ratio in lipids of ruminants is often regarded as too high (Aurousseau et al. 2004). The n‐6:n‐3 ratio of beef and lamb loin steaks was, however, determined as 2.11 and 1.32 respectively (Wood et al. 2003). Lestingi et al. (2015) recorded n‐6:n‐3 ratios as low as 3.28–4.01 in *longissimus dorsi* muscle of lambs fed diets containing faba and lupin seeds.

Following an increasing ADF content in the current study, the n‐6:n‐3 ratio of subcutaneous lipid varied between 11.32 and 9.46 (Table 4), which was higher than the preferred ratio. Treatment ADF3 came the closest to the preferred value. The higher the ADF content of lamb finishing diets in relation to the low NDF content resulted in a more desirable n‐6:n‐3 ratio, even though far from satisfactory. The recommended ratios proposed by Wood et al. (2003) and Webb and O'Neill (2008) still leave room for improvement. A lipid source low in n‐6 and higher in n‐3 content is proposed for improved results.

The atherogenicity index of both muscle (Table 3) and lipid (Table 4) tissue was unaffected by dietary treatment. Fat with a high atherogenicity index has negative human health implications (Manso et al. 2009), specifically increasing the risk of cardiovascular disease (Carvalho et al. 2014). Palmitic acid concentrations can be harmful to human health as it increases low‐density lipoprotein (LDL) levels in the blood (Carvalho et al. 2014). In the current study, both these FAs within the muscle and lipid tissue of lamb (Tables 3 and 4, respectively) were left unaffected by dietary treatment, which might explain the lack of the atherogenicity index effect.

Dietary ADF content had a limited effect on longer‐chain PUFA content (Tables 3 and 4). Only arachidonic acid (C20:4c5,8,11,14) of adipose tissue in ADF3 was higher than CON and ADF2 (Table 4). From the current study, it was viable to increase favorable FAs in lamb meat (especially that of adipose tissue) by incorporating a fiber source high in ADF in low fiber finishing diets of lambs.

### 3.2 Lamb Muscle Tissue Stability and Tenderness

Strategies that alter meat FA composition (amount and type) could influence meat quality, especially tenderness and flavor (Webb and O'Neill 2008). Other aspects include firmness, color, and stability (Wood et al. 2003). Color of red meat is the most important sensory attribute affecting consumer purchasing decisions (Abdalla Filho et al. 2017). The oxidation of lipids and pigments is closely related in beef, with the increase in one resulting in a similar increase in the other (Carvalho et al. 2014). The oxidation of myoglobin, in addition to meat lipids during storage, may also lead to changes in color and texture of meat (Hajji et al. 2016; Yagoubi et al. 2018). Myoglobin (deoxymyoglobin or oxymyoglobin) is oxidized to metmyoglobin due to decreased metmyoglobin reducing activity. This results in metmyoglobin accumulation in the meat and is caused by an increased susceptibility to lipid oxidation (PUFA) (Ladeira et al. 2014).

The FA composition was not affected by ADF content (Table 3). Hence, a limited response in muscle color and oxidative stability was expected. The effect of increasing ADF content on the color stability and tenderness muscle tissue is presented in Table 5. The ADF content had a limited effect on muscle color stability. On day two of storage, muscle lightness (L*‐value) was higher for ADF3 compared to CON. Day eight showed an increased color stability in ADF1 compared to CON with the most change in yellowness (b*‐value) and Hue angle. No meaningful conclusion from the data presented in Table 5 could be drawn as ADF content seemed to have very little effect on meat color stability.

**TABLE 5:** The effect of increasing acid detergent fiber content in finishing diets on the color stability and tenderness of South African Merino mutton *Longissimus thoracis* muscle tissue.

Parameter	Treatment means (±SD)				*p*	CV (%)
	CON	ADF1	ADF2	ADF3		
**Day 0**						
L*	40.68 (1.11)	41.49 (1.51)	41.59 (2.23)	41.54 (2.98)	0.5889	5.04
a*	17.72 (1.53)	18.54 (1.72)	17.77 (1.07)	18.40 (1.38)	0.2908	7.99
b*	6.26 (0.95)	6.89 (0.93)	6.38 (0.77)	6.72 (1.03)	0.2292	14.16
Chroma	18.81 (1.73)	19.80 (1.87)	18.90 (1.23)	19.60 (1.61)	0.2595	8.48
Hue angle (degree)	19.32 (1.48)	20.29 (1.57)	19.69 (1.52)	19.94 (1.75)	0.4003	7.99
**Day 2 (storage at 4°C)**						
L*	48.53^b^ (1.61)	49.77^ab^ (1.55)	50.14^ab^ (1.91)	50.87^a^ (1.88)	0.0052	3.50
a*	13.48 (1.18)	12.62 (1.32)	12.82 (0.89)	12.78 (0.99)	0.1658	8.61
b*	8.51 (0.62)	8.69 (0.57)	8.86 (0.79)	8.56 (0.68)	0.4927	7.69
Chroma	15.96 (1.13)	15.34 (1.26)	15.60 (0.97)	15.41 (0.97)	0.4087	7.00
Hue angle (degree)	32.31 (2.57)	34.65 (2.57)	34.66 (2.55)	33.77 (2.70)	0.0555	7.68
**Day 4 (storage at 4°C)**						
L*	48.54 (1.67)	49.31 (1.62)	50.11 (2.16)	49.99 (2.10)	0.1061	3.84
a*	12.28 (1.23)	11.36 (1.12)	11.56 (1.19)	11.83 (1.48)	0.2258	10.74
b*	8.59 (0.84)	8.83 (0.65)	9.06 (0.83)	8.74 (0.96)	0.5042	9.38
Chroma	15.03 (1.08)	14.41 (1.02)	14.72 (1.03)	14.75 (1.41)	0.5401	7.79
Hue angle (degree)	35.07 (3.95)	37.87 (3.12)	38.14 (3.92)	36.53 (4.14)	0.1204	10.30
**Day 6 (storage at 4°C)**						
L*	48.40 (2.05)	49.31 (2.32)	49.47 (3.02)	50.11 (2.06)	0.2825	4.83
a*	9.82 (1.59)	9.36 (1.21)	9.51 (1.25)	9.59 (1.43)	0.8342	14.44
b*	8.31 (1.02)	8.94 (1.04)	8.88 (1.15)	8.17 (1.19)	0.1501	12.84
Chroma	12.92 (1.51)	12.99 (1.21)	13.06 (1.27)	12.70 (1.11)	0.8826	9.93
Hue angle (degree)	40.45 (4.97)	43.67 (4.58)	43.07 (5.03)	40.52 (6.82)	0.2439	12.95
**Day 8 (storage at 4°C)**						
L*	48.10 (2.05)	49.49 (1.89)	49.25 (3.27)	49.54 (2.74)	0.3743	5.16
a*	9.28 (1.38)	8.18 (0.90)	9.29 (1.98)	9.34 (1.51)	0.1057	16.41
b*	7.21^b^ (1.34)	8.94^a^ (1.12)	8.24^ab^ (1.83)	7.75^ab^ (1.45)	0.0143	18.07
Chroma	11.91 (0.62)	12.20 (0.69)	12.60 (1.76)	12.28 (1.32)	0.4807	9.64
Hue angle (degree)	38.07^b^ (8.84)	47.38^a^ (5.95)	41.62^ab^ (9.49)	39.76^ab^ (7.73)	0.0158	19.39
**Tenderness**						
Warner‐Bratzler shear force (kg)	2.62^ab^ (0.60)	2.83^a^ (0.74)	2.22^b^ (0.45)	2.26^ab^ (0.61)	0.0240	24.54

*Note:* The ADF content of diet dry matter (DM) for respective treatments are CON = 46.8 g/kg, ADF1 = 59.3 g/kg, ADF2 = 63.8 g/kg, ADF3 = 79.9 g/kg; L*: Lightness; a*: Redness; b*: Yellowness; Chroma: Saturation index. ^a,b^Mean values with different superscripts in the same row differ significantly (*p* < 0.05).

Abbreviations: ADF, acid detergent fiber; CON, control; CV, coefficient of variation.

The susceptibility of muscle tissue to lipid oxidation depends on PUFA levels (Buckley et al. 1995) and its deterioration is triggered by a higher UFA content (Vatansever et al. 2000). Increased oxidative breakdown during the post‐mortem storage period reduces shelf life (Vatansever et al. 2000). The lack of dietary response regarding overall meat color stability (Table 5) is therefore associated with the same lack of treatment effect where muscle FA composition is concerned (Table 3).

A subcutaneous b*‐value of 22 or more in lamb carcasses can easily be distinguished with the naked eye (Priolo et al. 2002). The b*‐value in all ADF treatments and storage times, were much lower than 22 (Table 5). According to Priolo et al. (2002), the yellow color of ruminant meat (b*‐value) is related to the presence of carotenoids normally contained at higher levels in grasses.

Hue angle gives a more realistic perspective on meat browning than any other single parameter (Luciano et al. 2009). The closer the Hue angle is to 90^o^, the more the color corresponds to yellow (Priolo et al. 2002). All values measured for ADF treatment and storage time were also lower than the recommended value (Table 5).

Meat from ADF2 was more tender than ADF1 (Table 5). Presenting any plausible cause for this result is difficult. No consistent effect was noted with the increase in ADF content. Limited literature regarding dietary fiber type and quality effect on meat tenderness was found.

The effect of increasing ADF content in low fiber finishing diets on oxidative stability (malonaldehyde content) of lamb muscle tissue is presented in Table 6.

**TABLE 6:** The effect of increasing acid detergent fiber of finishing diets on the malonaldehyde content of South African Merino mutton muscle tissue.

Thiobarbituric acid reactive substances (mg malonaldehyde/kg muscle tissue)	Treatment diet means (±SD)				*p*	CV (%)
	CON	ADF1	ADF2	ADF3		
Day 0 (refrigerated storage at 4°C)	0.05 (0.03)	0.06 (0.02)	0.05 (0.02)	0.05 (0.02)	0.5334	42.69
Day 8 (refrigerated storage at 4°C)	0.43^b^ (0.28)	0.76^a^ (0.40)	0.46^b^ (0.26)	0.44^b^ (0.25)	0.0105	58.58
Day 90 (frozen storage at −18°C)	0.11 (0.04)	0.13 (0.07)	0.12 (0.06)	0.11 (0.04)	0.4630	46.27

*Note:* The ADF content of diet dry matter (DM) for respective treatments are CON = 46.8 g/kg, ADF1 = 59.3 g/kg, ADF2 = 63.8 g/kg, ADF3 = 79.9 g/kg. ^a,b^Mean values with different superscripts in the same row differ significantly (*p* < 0.05).

Abbreviations: ADF, acid detergent fiber; CON, control; CV, coefficient of variation.

From Table 6 it is evident that no significant treatment effect occurred for meat samples tested on Day 0 after refrigerated storage (4°C) or stored for 90 days at −18°C. In a study by Zhang et al. (2019) the malonaldehyde content of *M. longissimus lumborum* sampled from grass‐fed beef ranged from 0.04 to 10.72 mg malonaldehyde/kg, with the majority of the data < 1.0 mg malonaldehyde/kg. The ADF1 (59.3 g/kg) had an increased malonaldehyde content compared to the other treatments when stored for 8 days at 4°C. No meaningful conclusions were drawn because of lack of ADF effect on the malonaldehyde content.

Scollan et al. (2006) explained that the stability of meat is more related to FA composition than antioxidant concentration. Changing the FA profile of meat to more UFA increases oxidative breakdown during the post‐mortem display (Vatansever et al. 2000; Ladeira et al. 2014), which makes meat preservation more difficult (Ladeira et al. 2014). Meat with additional PUFAs is more susceptible to lipid oxidation than SFAs (Yagoubi et al. 2018). The intensity of muscle oxidation has therefore a positive correlation with the degree of UFA concentration and its interface with oxygen (Carvalho et al. 2014). The increase in TBARS over retail display time may also be related to myoglobin oxidation which produces free radicals (i.e., haem‐ions and/or haem‐proteins) that initiate and promote lipid oxidation (Uushona et al. 2023). The primary reaction of oxygen with a saturated hydrocarbon chain is slow, but increases in the presence of double bonds. Linolenic acid with its three double bonds is oxidized 100 times more rapidly than oleic acid with only one double bond (Enser 1984). Monounsaturated fatty acids are therefore more resistant to oxidative modification than PUFAs (McDonald et al. 2011). The only significant effect on muscle FA composition was that of α‐linolenic acid and CLAc9,t11 (Table 3). Therefore, due to a lack of response in overall lamb muscle FA composition, no effect was expected, and no definitive conclusion was drawn in this regard. The CLA derivates of ruminant products are relatively stable during storage (Bauman et al. 1999) and has antioxidant activity (in meat) (Hur et al. 2017). Although an increase in the n‐3 PUFA concentration is desirable from a human health perspective, the oxidative stability of meat is reduced (Hajji et al. 2016).

A malonaldehyde level of 0.5 mg/kg and higher is considered the turning point from where lipid oxidation causes rancid odor and taste of meat (Wood et al. 2008). This threshold was only exceeded by treatment ADF1 stored for eight days (Table 6). The applicable cut‐off value of malonaldehyde content per kg of meat is 2 mg (Vatansever et al. 2000; Wood et al. 2003). According to Ripoll et al. (2011), this accepted threshold value seems to be 1 mg malonaldehyde/kg meat.

The redness index (a*) of meat is associated with the instability of haeme pigments in secondary products (α‐ and β‐aldehydes) of lipid oxidation, causing the decreased stability of oxymyoglobin redox. Changes in a* and oxymyoglobin values appear to be driven by lipid oxidation and are strongly correlated with malonaldehyde values (Ladeira et al. 2014). Even though only the malonaldehyde of muscle tissue of lambs fed ADF1 was higher at eight days of refrigerated storage (Table 6), the lack of effect (*p* = 0.1057; Table 5) regarding the redness index of the same cuts seems to support this premise.

## 4 Conclusion

The FA composition of SAMM wether subcutaneous adipose tissue was significantly affected by dietary ADF content with an increase in unsaturated content, but with a limited effect on muscle tissue. A higher dietary ADF content compared to lower ADF fiber finishing diets with similar NDF increased subcutaneous lipid tissue n‐3 (α‐linolenic and total n‐3) and CLA c9,t11 content of lamb, with a concomitant decrease (favorable) n‐6:n‐3 ratio. The n‐6:n‐3 ratio was, however, higher compared to the proposed threshold of 4:1. A dietary lipid source low in n‐6 and high in n‐3 content along with a high diet ADF content is proposed for increased subcutaneous lipid tissue n‐3 of lamb. The research should be repeated with other breeds of sheep to test this theory.

## Author Contributions

Ockert Bernard Einkamerer, Abraham Vlok Ferreira, and Arnold Hugo designed the experiment and Ockert Bernard Einkamerer carried out the research trial. Michael Denis Fair completed the statistical analyses. Ockert Bernard Einkamerer and Arnold Hugo structured the scientific content and Ockert Bernard Einkamerer drafted the manuscript. Abraham Vlok Ferreira and Arnold Hugo assisted with the experimental design and research trial, while all authors provided editorial suggestions and approved the final manuscript. Arnold Hugo conducted the meat quality analysis.

## Funding

This study was funded by the Directorate Research and Development of the University of the Free State, Bloemfontein, South Africa.

## Conflicts of Interest

The authors declare no conflicts of interest.

## Data Availability

The datasets generated during and/or analyzed during the current study are not publicly available due to intellectual property but are available from the corresponding author on reasonable request.
